# Net water uptake combined with neutrophil-to-lymphocyte ratio predictive value after successful recanalization in acute large vessel occlusion stroke

**DOI:** 10.3389/fneur.2025.1633967

**Published:** 2025-10-13

**Authors:** Xu Jing, Xiang Liangxu, Li Zhide, Zhao Yue, Tian Yanghua

**Affiliations:** ^1^Department of Neurology, Ma'anshan People's Hospital, Maanshan, Anhui, China; ^2^Anhui Medical University, Hefei, Anhui, China

**Keywords:** endovascular thrombectomy, acute large vessel occlusion stroke, net water uptake, neutrophil-to-lymphocyte ratio, poor outcome

## Abstract

**Objective:**

Despite successful recanalization after endovascular thrombectomy (EVT), some patients with acute large vessel occlusion stroke (ALVOS) have poor clinical outcomes. This study employed net water uptake (NWU) which was calculated based on the cranial CT on admission, to investigate the factors associated with the clinical outcomes of ALVOS patients with successful EVT recanalization.

**Methods:**

ALVOS patients in anterior circulation with successful EVT recanalization were consecutively enrolled. NWU was measured in the middle cerebral artery territory based on the preoperative cranial CT, calculated by (1 − affected hemisphere density/ contralateral hemisphere density) × 100%. The neutrophil-to-lymphocyte ratio (NLR) was calculated from the blood routine test on admission. A poor 90-day outcome was defined as a modified Rankin Scale (mRS) > 2 points at 90-day after the index stroke.

**Results:**

A total of 113 participants were enrolled. NLR (odds ratio [OR] = 1.31, 95% confidence interval [CI] = 1.09–1.58, *p* = 0.004) and NWU (OR = 1.48, 95% CI = 1.21–1.81, *p* < 0.001) were independently associated with poor 90-day outcomes. In the restricted cubic spline analysis, a significant nonlinear relationship was observed between NWU and an increased risk of 90-day poor functional outcome (*p* for nonlinear = 0.018). All participants were categorized into three grades based on 90-day mRS: complete independence (mRS 0–1 point), partial dependence (mRS 2–3 points), and complete dependence or mortality (mRS 4–6 points). In the multivariate ordinal logistic regression, both NLR (OR = 1.32, 95% CI = 1.12–1.56, *p* = 0.001) and NWU (OR = 1.29, 95% CI = 1.10–1.51, *p* = 0.002) were independently associated with the 90-day functional outcome grade. Receiver operating characteristic analysis demonstrated that the combination of NWU and NLR had the highest indicative value of poor outcome (area under the curve [AUC] = 0.800, 95% CI = 0.718–0.881, *p* < 0.001), followed by sole NWU (AUC = 0.764, 95% CI = 0.674–0.855, *p* < 0.001) and NLR (AUC = 0.662, 95% CI = 0.563–0.762, *p* = 0.003).

**Conclusion:**

The combination of NWU and NLR provides stronger indicative value of poor outcome compared to either marker alone.

## Introduction

Acute large vessel occlusion stroke (ALVOS) is a severe subtype of ischemic stroke, for which endovascular thrombectomy (EVT) has emerged as the first-line treatment (1). Although EVT significantly reduces the 90-day disability risk in ALVOS patients (2), a substantial number of patients still experience poor clinical outcomes (3), particularly those with large core infarctions (4–6). For ALVOS patients undergoing EVT, successful recanalization is one of the key determinants of functional outcome (7, 8). Currently, researches investigating the clinical factors associated with clinical outcomes of ALVOS patients with successful EVT recanalization are rare. A recent study reported that postoperative 7-day NIHSS (National Institute of Health Stroke Scale) score, neutrophil-lymphocyte ratio (NLR), and pre-existing diabetes may be associated with 90-day clinical outcomes in successfully recanalized anterior circulation ALVOS patients (8).

Net water uptake (NWU) is an emerging CT-based biomarker developed in recent years, and it is calculated by comparing Hounsfield unit values between the infarcted region and contralateral brain parenchyma on non-contrast CT or CT perfusion imaging (9). NWU reflects the degree of early ischemic tissue hypoattenuation and is directly proportional to early edematous water uptake. Currently, NWU is primarily used to predict the occurence of malignant edema after EVT (10–12), which is one of the determinants of clinical outcomes in ALVOS patients after EVT (13). After the onset of ischemic stroke, osmotically active solutes rapidly shift from the interstitial compartment into intracellular spaces, triggering cytotoxic edema through disruption of ionic gradients in the blood–brain barrier and subsequent water influx. Subsequent degradation of endothelial tight junction proteins (e.g., occludin, claudin-5) mediated by matrix metalloproteinase-9 activation triggers blood–brain barrier breakdown, facilitating vasogenic edema formation that contributes to progressive NWU elevation. Consequently, elevated preoperative NWU quantified on admission CT is closely correlated with endothelial injury and blood-brain barrier disruption (14). This may represent the underlying mechanism by which NWU is associated with adverse functional outcomes in patients with ischemic stroke. A recent study confirmed that NWU predicts 90-day functional outcomes after thrombectomy in ALVOS patients with an ASPECT score of ≤ 5 (15). However, this study only included subjects with low ASPECT scores, for whom thrombectomy is not strongly recommended. It remains unclear whether this metric can still predict 90-day functional outcomes in patients with ASPECT scores above 6 points who are indicated for thrombectomy.

Neutrophil-to-lymphocyte ratio (NLR), as an inflammatory marker, has been demonstrated to be associated with the severity of inflammation, hemorrhagic transformation, and poor clinical outcomes in cerebral infarction (16, 17). A recent retrospective study reported that the NLR was independently associated with 90-day functional outcomes in anterior ALVOS patients with successful EVT recanalization (18). However, some researchers argue that NLR is susceptible to be influenced by concurrent infections or systemic inflammation in patients (19). Therefore, we suggest that relying solely on NLR may not be sufficiently reliable for predicting functional outcomes of ALVOS patients.

In this study, we enrolled ALVOS patients with successful EVT recanalization, and employed NWU and NLR as one of the observational parameters, aiming to explore clinical factors associated with 90-day clinical outcomes in these patients, and to early identify successfully recanalized ALVOS patients with high risk of poor 90-day outcomes in clinical practice.

## Methods

### Study population

This study consecutively enrolled patients diagnosed with anterior circulation ALVOS who achieved successful recanalization after mechanical thrombectomy at Ma’anshan People’s Hospital between January 2022 and December 2023. This study was approved by the hospital Institutional Ethics Committee, and written informed consents were obtained from all participants’ legal guardians.

The inclusion criteria were as follows: (1) age >18 years; (2) the occlusion of the internal carotid artery or middle cerebral artery was confirmed by computed tomography angiography or digital subtraction angiography; (3) with symptom onset within 6 h, or with onset within 24 h in case of fulfilling DEFUSE-3 or DAWN criteria on computed tomography perfusion (20, 21); (4) with an Alberta Stroke Program Early CT Score ≥ 6 points on pre-procedural imaging; (5) with a pre-procedural NIHSS score ≥ 6 points; (6) with a pre-procedural modified Rankin Scale (mRS) < 2 points. Patients would be excluded if: (1) without neuroimaging evidence after EVT; (2) with post-procedural modified Thrombolysis in Cerebral Infarction (mTICI) grade < 2b; (3) with comorbidities including chronic obstructive pulmonary disease, severe cardiac conditions, hematologic disorders, or active malignancies; (4) with a pre-stroke mRS > 2 points.

### Baseline data

Upon the admission to the emergency room, the baseline demographic and clinical characteristics of each participant was collected through a face-to-face interview conducted by a neurologist, including age, sex, history of hypertension, diabetes mellitus, previous stroke and atrial fibrillation, and smoking status. The severity of neurological deficits of participants was evaluated according to the NIHSS score. The daily activity of participants before the index stroke was assessed by a questionnaire based on the mRS score. All participants underwent blood routine and coagulation routine tests before EVT, the neutrophil and lymphocyte counts were acquired and the NLR was calculated.

### The evaluation of NWU

**Figure 1:**
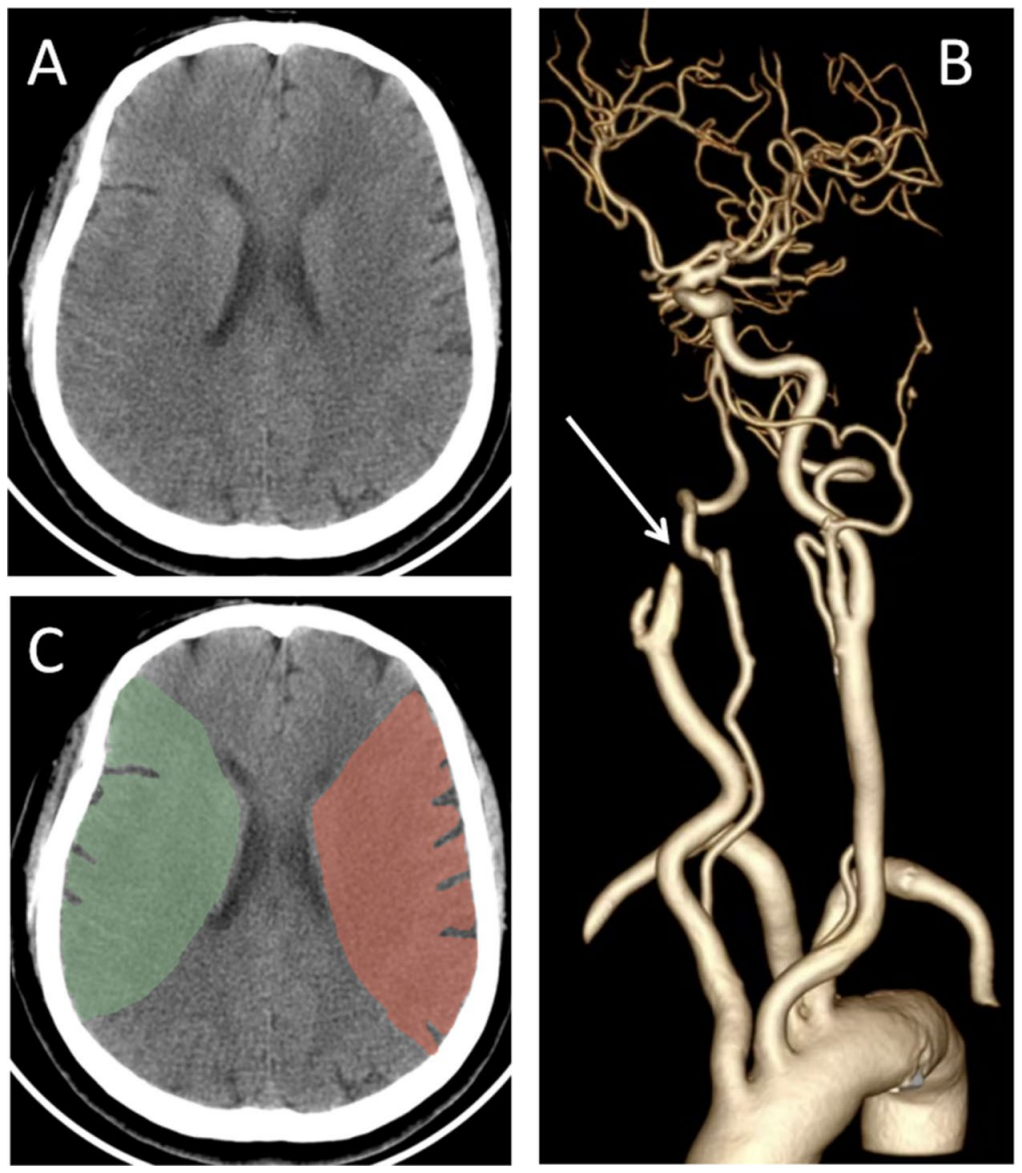
Representative cases of the NWU measurement. **(A)** The preoperative cranial CT of a ALVOS patient; **(B)** the CT angiography showed the occlusion of the right internal carotid artery; **(C)** the NWU was calculated by (1- density of affected territory[green region]/density of contralateral territory[red region]) × 100%. ALVOS indicates acute large vessel occlusion stroke; NWU, net water uptake.

Before EVT, all participants underwent cranial CT, CT angiography and CT perfusion imaging, and underwent follow-up cranial CT scans at 24 h, 3 days, and 7 days after EVT. Additional cranial CT scans were performed immediately if participants developed deterioration in consciousness or clinical neurological status. Based on the admission cranial CT imaging, the NWU in the affected middle cerebral artery territory was measured by using the Segment Editor module of 3D Slicer software. Firstly, the bilateral middle cerebral artery territories were delineated. The anterior boundary was defined by the extension line of the anterior horn of the lateral ventricle, while the posterior boundary was defined by the extension line of the posterior horn of the lateral ventricle. The outer boundary followed the cerebral cortex margin, and the inner boundary was marked by the medial edge of the caudate nucleus and the posterior limb of the internal capsule (Figure 1). Subsequently, the Radiomics module was employed to automatically acquire the parenchymal density values of bilateral middle cerebral artery territories. The NWU was calculated by (1- density of affected territory/density of contralateral territory) × 100% (22).

### Administration strategy

For participants with onset within the intravenous thrombolysis time window and with the indications for intravenous thrombolysis, intravenous thrombolysis bridging EVT was administered in case of consents provided from their legal guardians. If the participants with the onset exceeding 6-h intravenous thrombolysis time window met the DEFUSE-3 or DAWN criteria, EVT will be performed with consents from the legal guardians. The surgical procedures of EVT included aspiration thrombectomy, stent-retriever thrombectomy, and (or) emergency stenting. The EVT procedures were performed under general anesthesia. After successful femoral artery sheath placement, comprehensive cerebral angiography was performed to identify the target occluded vessel. Under the guidance of a microguidewire (Avigo, EV3), a microcatheter (Rebar 18, EV3) was then advanced beyond the vascular occlusion site. A subsequent angiography was performed to verify whether the microcatheter was placed within the true vascular lumen. Depending on the participant’s specific condition, either pure aspiration thrombectomy or a combined approach of aspiration with stent-retriever (Solitare AB/FR, EV3) thrombectomy was subsequently employed. The operator recorded the following parameters: door-to-puncture time, time to recanalization, number of retrieval attempts and emergency stent placement.

After the removal of thrombus, the status of recanalization was evaluated by using the modified Thrombolysis in Cerebral Infarction grading system, and the successful vascular recanalization was defined as an mTICI grade of 2b or 3 (23). All participants underwent postprocedural cranial CT to assess for hemorrhage. For patients receiving bridging therapy (intravenous thrombolysis followed by EVT), antithrombotic agents and statins were initiated 24 h after thrombolysis. In those treated with EVT alone, antithrombotic and statin therapies were administered immediately after the procedure. The postoperative blood pressure of all participants was under intensive control to prevent intracranial hyperperfusion. For participants presenting with cerebral edema, dehydration therapy was administered.

### Follow-up

An independent neurologist blinded to the baseline data conducted the telephone interviews with the participants or their legal guardians at 30, 60, and 90 days after the index stroke. Standardized questionnaires were administered to evaluate the control of vascular risk factors and medication adherence during follow-up. At the 90-day follow-up, the clinical outcomes were evaluated according to the mRS (0–5 points, death was recorded as 6 points). A poor clinical outcome was defined as a mRS > 2 points.

### Statistical analysis

At first, we completed the missing data by using multiple imputation (5 imputations) and chose the set of data with the highest Cronbach’s alpha coefficient in reliability analysis. The normality of continuous variables was assessed by using the Shapiro–Wilk test. Normally distributed continuous variables were described by mean ± standard deviation, while non-normally distributed variables were presented as median (interquartile range). Categorical variables were compared by using the chi-square test. For normally distributed continuous variables, independent samples *t*-test and one-way analysis of variance test were employed for comparisons between two groups and multiple groups, respectively. For non-normally distributed continuous variables, the Mann–Whitney *U* test and Kruskal-Wallis test were utilized for comparisons between two groups and multiple groups, respectively.

Clinical factors with *p* < 0.05 in univariate analyses were included in a binary multivariable logistic regression model to identify factors independently associated with a 90-day mRS > 2 points of participants. Restricted cubic splines were used to analyze the potential nonlinear association between NWU, NLR, and a 90-day mRS > 2 points. Based on the mRS scores at 90 days, functional outcomes of all participants were categorized into three grades: complete independence (mRS 0–1 point), partial dependence (mRS 2–3 points), and complete dependence or mortality (mRS 4–6 points). Ordinal logistic regression was used to analyze the associations between clinical factors and the ordinal grades of 90-day mRS scores. Receiver operating characteristic (ROC) curves were generated to evaluate the indicative value of each factor for a 90-day mRS > 2 points, quantified by the area under the curve (AUC). All statistical tests were two-tailed, and *p* < 0.05 was considered statistically significant. Restricted cubic splines was performed by using R software (version 4.3.0, package plotRCS). Other statistical analyses were performed by using SPSS 25.0 (IBM, Armonk, NY, USA).

## Results

**Figure 2:**
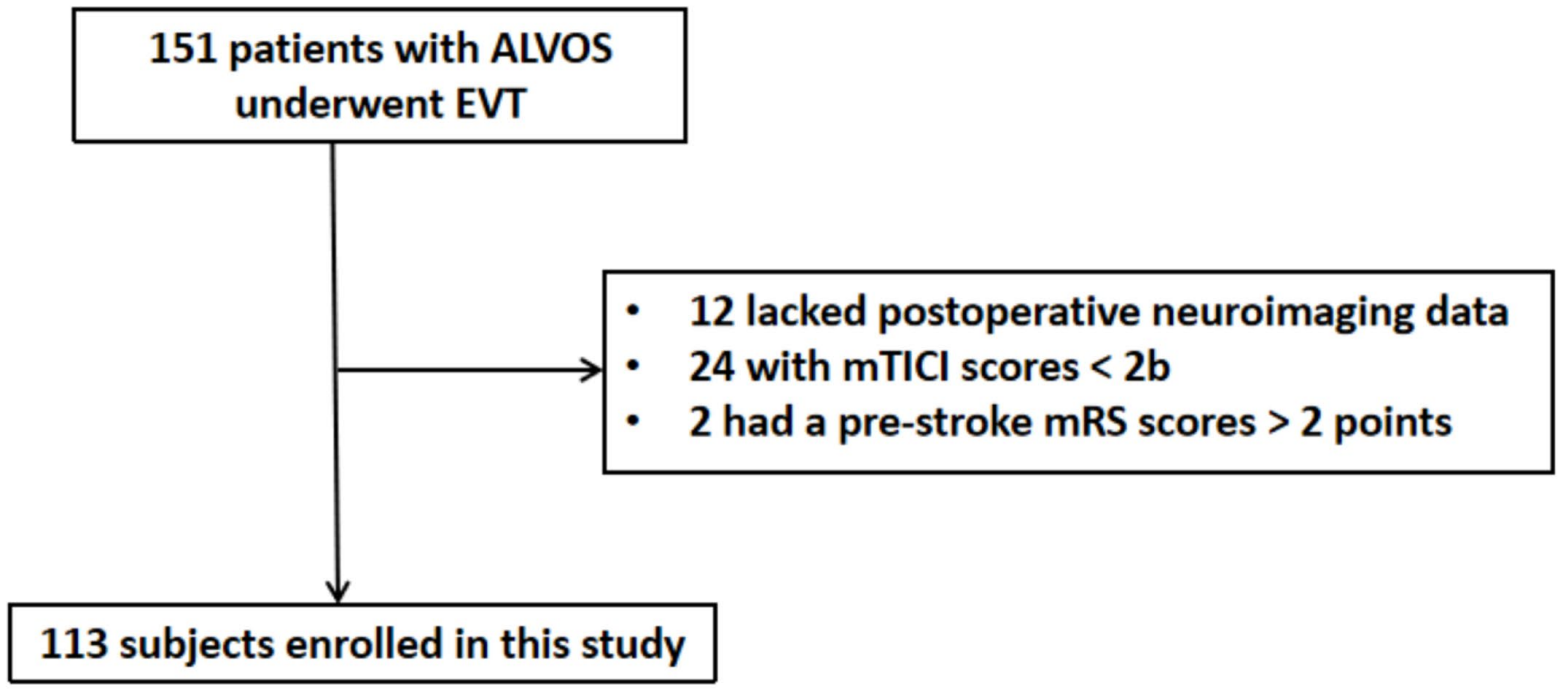
Flow diagram of subjects’ enrollment. ALVOS indicates acute large vessel occlusion stroke; EVT, endovascular thrombectomy; mTICI, modified Thrombolysis in Cerebral Infarction; mRS, modified Rankin Scale.

A total of 151 patients with ALVOS underwent EVT. Of these patients, 12 lacked postoperative neuroimaging data, 24 had a postoperative mTICI scores < 2b, and 2 had a pre-stroke mRS scores > 2 points. These patients were all excluded (Figure 2). Consequently, 113 patients were included in the final analysis. The cohort comprised 62 (54.9%) males, with a median age of 70.0 (58.0–76.5) years.

### Baseline data

The mean NIHSS score on admission was 15.3 ± 3.6 points. Intravenous thrombolysis was administered to 34 (30.1%) patients. The internal carotid artery of 85 (75.2%) patients and the M1-M2 segments of the middle cerebral artery of 28 (24.8%) patients were occlusive. Based on preoperative cranial CT imaging, the mean NWU was 7.72 ± 2.74%. The median door-to-puncture time DPT was 109.0 (83.0–137.8) minutes, with a median of 2.0 (1.0–2.0) retrieval attempts. Emergency stenting was performed in 14 (12.4%) patients. The median recanalization time was 152.0 (132.8–195.0) minutes. Among the 113 participants, 60 (53.1%) had poor 90-day clinical outcomes, and 8 (7.1%) died (Table 1).

**Table 1:** The baseline clinical characteristics of all participants.

Clinical characteristics	Total (*n* = 113)
Male, *n* (%)	62 (54.9)
Age (year), median (IQR)	70 (58.0, 76.5)
Hypertension, *n* (%)	96 (85.0)
Diabetes mellitus, *n* (%)	29 (25.7)
Atrial fibrillation, *n* (%)	52 (46.0)
Smoking, *n* (%)	26 (23.0)
Previous stroke, *n* (%)	35 (31.0)
Initial NIHSS (pioint), mean ± SD	15.3 ± 3.6
SBP (mmHg), mean ± SD	149.96 ± 23.88
DBP (mmHg), median (IQR)	80.0 (70.0, 90.0)
Intravenous thrombolysis, *n* (%)	34 (30.1)
TC (mmol/L), mean ± SD	3.96 ± 0.96
TG (mmol/L), median (IQR)	0.92 (0.72, 1.23)
LDL-C (mmol/L), mean ± SD	2.41 ± 0.86
HDL-C (mmol/L), mean ± SD	1.26 ± 0.30
BUN (mmol/L), median (IQR)	6.25 (5.02, 7.07)
Cr (mmol/L), median (IQR)	70.0 (59.3, 83.3)
Uric acid (mmol/L), median (IQR)	336.9 (265.3, 402.5)
HCY (mmol/L), median (IQR)	9.95 (8.28, 12.13)
HbA1c (%), median (IQR)	5.90 (5.50, 6.50)
Serum lean protein (g/L), mean ± SD	41.45 ± 3.45
Lymphocyte count (×10^9^/L), mean ± SD	1.57 ± 0.79
Platelet count (×10^9^/L), mean ± SD	159.00 ± 70.71
NLR, median (IQR)	3.34 (2.13, 6.60)
NWU, mean ± SD	7.72 ± 2.74
DPT (min), median (IQR)	109.0 (83.0, 137.8)
Occlusion site, *n* (%)	
ICA	85 (75.2)
MCA trunk (M1-M2)	28 (24.8)
Number of retrieval attempts, median (IQR)	2.0 (1.0, 2.0)
Implant stent, *n* (%)	14 (12.4)
Recanalization time (min), median (IQR)	152.0 (132.8, 195.0)
Poor outcome, *n* (%)	60 (53.1)

NIHSS indicates National Institutes of Health Stroke Scale; SBP, systolic blood pressure; DBP, diastolic blood pressure; TC, total cholesterol; TG, triglyceride; LDL-C, low-density lipoprotein cholesterol; HDL-C, high-density lipoprotein cholesterol; Hcy, homocysteine; HbA1c, glycosylated hemoglobin; NLR, neutrophil-lymphocyte ratio; NWU, net water uptake; DPT, door-to-puncture time; ICA, internal carotid artery; MCA, middle cerebral artery.

### Comparisons between participants with good and poor outcomes

Compared to ALVOS patients with good outcomes after successful EVT recanalization, those with poor outcomes exhibited significantly higher preoperative systolic blood pressure (157.2 ± 24.6 vs. 141.8 ± 20.4 mmHg, *p* < 0.001) and diastolic blood pressure [85.0 (75.3–94.5) vs. 78.0 (69.5–88.5) mmHg, *p* = 0.011], NLR [4.41 (2.25–8.76) vs. 2.57 (1.55–4.70), *p* = 0.003] and NWU (8.81 ± 2.68 vs. 6.48 ± 2.25, *p* < 0.001) (Table 2).

**Table 2:** The comparisons between participants with good and poor outcomes.

Clinical characteristics	Good outcome (*n* = 53)	Poor outcome (*n* = 60)	*p* value
Male, *n* (%)	32 (60.4)	30 (50.0)	0.27
Age (year), median (IQR)	68.0 (54.5, 77.0)	72.0 (63.0, 76.0)	0.14
Hypertension, *n* (%)	44 (83.0)	52 (86.7)	0.59
Diabetes mellitus, *n* (%)	11 (20.8)	18 (30.0)	0.26
Atrial fibrillation, *n* (%)	21 (39.6)	31 (51.7)	0.20
Smoking, *n* (%)	10 (18.9)	16 (26.7)	0.33
Previous stroke, n (%)	13 (24.5)	22 (36.7)	0.16
Initial NIHSS (pioint), mean ± SD	14.7 ± 3.6	15.8 ± 3.6	0.12
SBP (mmHg), mean ± SD	141.8 ± 20.4	157.2 ± 24.6	<0.001^*^
DBP (mmHg), median (IQR)	78.0 (69.5, 88.5)	85.0 (75.3, 94.5)	0.011^*^
Intravenous thrombolysis, *n* (%)	14 (26.4)	20 (33.3)	0.42
TC (mmol/L), mean ± SD	3.98 ± 0.82	3.95 ± 1.08	0.88
TG (mmol/L), median (IQR)	0.98 (0.72, 1.28)	0.89 (0.71, 1.18)	0.48
LDL-C (mmol/L), mean ± SD	2.43 ± 0.78	2.38 ± 0.92	0.79
HDL-C (mmol/L), mean ± SD	1.26 ± 0.31	1.26 ± 0.30	0.97
BUN (mmol/L), median (IQR)	5.52 (5.03, 7.04)	6.58 (4.89, 7.22)	0.17
Cr (mmol/L), median (IQR)	72.40 (60.68, 82.60)	68.50 (56.55, 83.65)	0.36
Uric acid (mmol/L), median (IQR)	334.90 (232.40, 395.93)	338.70 (280.25, 404.15)	0.58
HCY (mmol/L), median (IQR)	9.50 (8.10, 11.50)	10.30 (8.45, 12.90)	0.38
HbA1c (%), median (IQR)	5.80 (5.40, 6.15)	6.00 (5.60, 6.90)	0.051
Serum lean protein (g/L), mean ± SD	41.48 ± 3.02	41.43 ± 3.82	0.95
Lymphocyte count (×10^9^/L), mean ± SD	1.64 ± 0.63	1.51 ± 0.92	0.39
Platelet count (×10^9^/L), mean ± SD	161.79 ± 53.13	176.64 ± 83.25	0.27
NLR, median (IQR)	2.57 (1.55, 4.70)	4.41 (2.25, 8.76)	0.003^*^
NWU, mean ± SD	6.48 ± 2.25	8.81 ± 2.68	<0.001^*^
DPT (min), median (IQR)	109.0 (78.5, 131.5)	109.0 (83.0, 140.0)	0.69
Occlusion site, *n* (%)			0.95
ICA	13 (24.5)	15 (25.0)	
MCA trunk (M1-M2)	40 (75.5)	45 (75.0)	
Number of retrieval attempts, median (IQR)	2.0 (1.0, 2.0)	2.0 (1.0, 2.0)	0.68
Implant stent, *n* (%)	7 (13.2)	7 (11.7)	0.80
Recanalization time (min), median (IQR)	148.0 (126.5, 182.5)	159.0 (142.0, 212.0)	0.15

NIHSS indicates National Institutes of Health Stroke Scale; SBP, systolic blood pressure; DBP, diastolic blood pressure; TC, total cholesterol; TG, triglyceride; LDL-C, low-density lipoprotein cholesterol; HDL-C, high-density lipoprotein cholesterol; Hcy, homocysteine; HbA1c, glycosylated hemoglobin; NLR, neutrophil-lymphocyte ratio; NWU, net water uptake; DPT, door-to-puncture time; ICA, internal carotid artery; MCA, middle cerebral artery.

*p < 0.05 was considered statistically significant.

### Factors independently associated with 90-day poor outcomes in ALVOS patients with successful EVT recanalization

Sex, age, and variables with significant differences in univariate analysis were incorporated into a binary multivariate logistic regression model. NLR (odds ratio [OR] = 1.31, 95% CI = 1.09–1.58, *p* = 0.004) and NWU (OR = 1.48, 95% CI = 1.21–1.81, *p* < 0.001) were independently associated with 90-day poor outcomes in ALVOS patients with successful EVT recanalization (Table 3).

**Table 3:** Clinical factors independently associated with poor outcomes in ALVOS patients with successful EVT recanalization.

Clinical factors	Poor outcome			
	Crude OR	*p*-value	Adjusted OR^†^	*p*-value
Male	1.52 (0.72–3.22)	0.27	1.96 (0.68–5.65)	0.21
Age	1.03 (1.00–1.07)	0.05	1.04 (0.99–1.08)	0.095
SBP	1.03 (1.01–1.05)	0.001^*^	1.02 (0.99–1.04)	0.25
DBP	1.04 (1.01–1.07)	0.013^*^	1.05 (0.997–1.10)	0.069
NLR	1.19 (1.06–1.34)	0.005^*^	1.31 (1.09–1.58)	0.004^*^
NWU	1.46 (1.22–1.75)	<0.001^*^	1.48 (1.21–1.81)	<0.001^*^

†Sex, age, SBP, DBP, NLR and NWU were included to the regression model simultaneously.

SBP indicates systolic blood pressure; DBP, diastolic blood pressure; NLR, neutrophil-lymphocyte ratio; NWU, net water uptake.

*p < 0.05 was considered statistically significant.

### The association of NWU and NLR with 90-day poor outcomes

**Figure 3:**
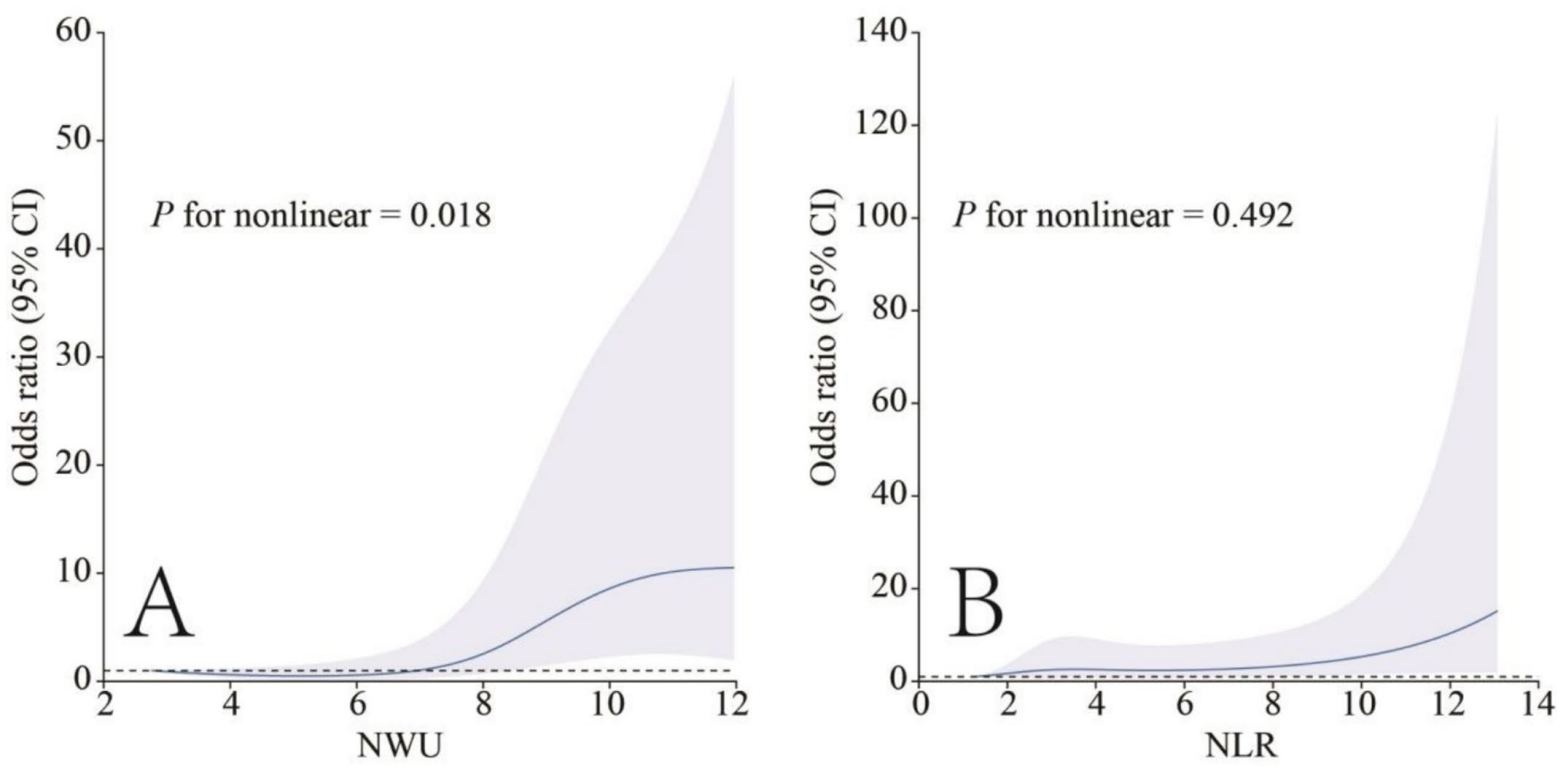
The relationship of NWU **(A)** and NLR **(B)** with 90-day poor outcomes in the restricted cubic spline analysis.

In the restricted cubic spline analysis, a significant nonlinear relationship was observed between NWU and an increased risk of 90-day poor functional outcome (*p* for nonlinear = 0.018). The risk of poor functional outcome at 90 days increased significantly when NWU > 8%. No nonlinear association was found between NLR and 90-day poor functional outcome at 90 days (*p* for nonlinear = 0.492) (Figure 3).

### Distribution of 90-day mRS scores across NWU and NLR levels

**Figure 4:**
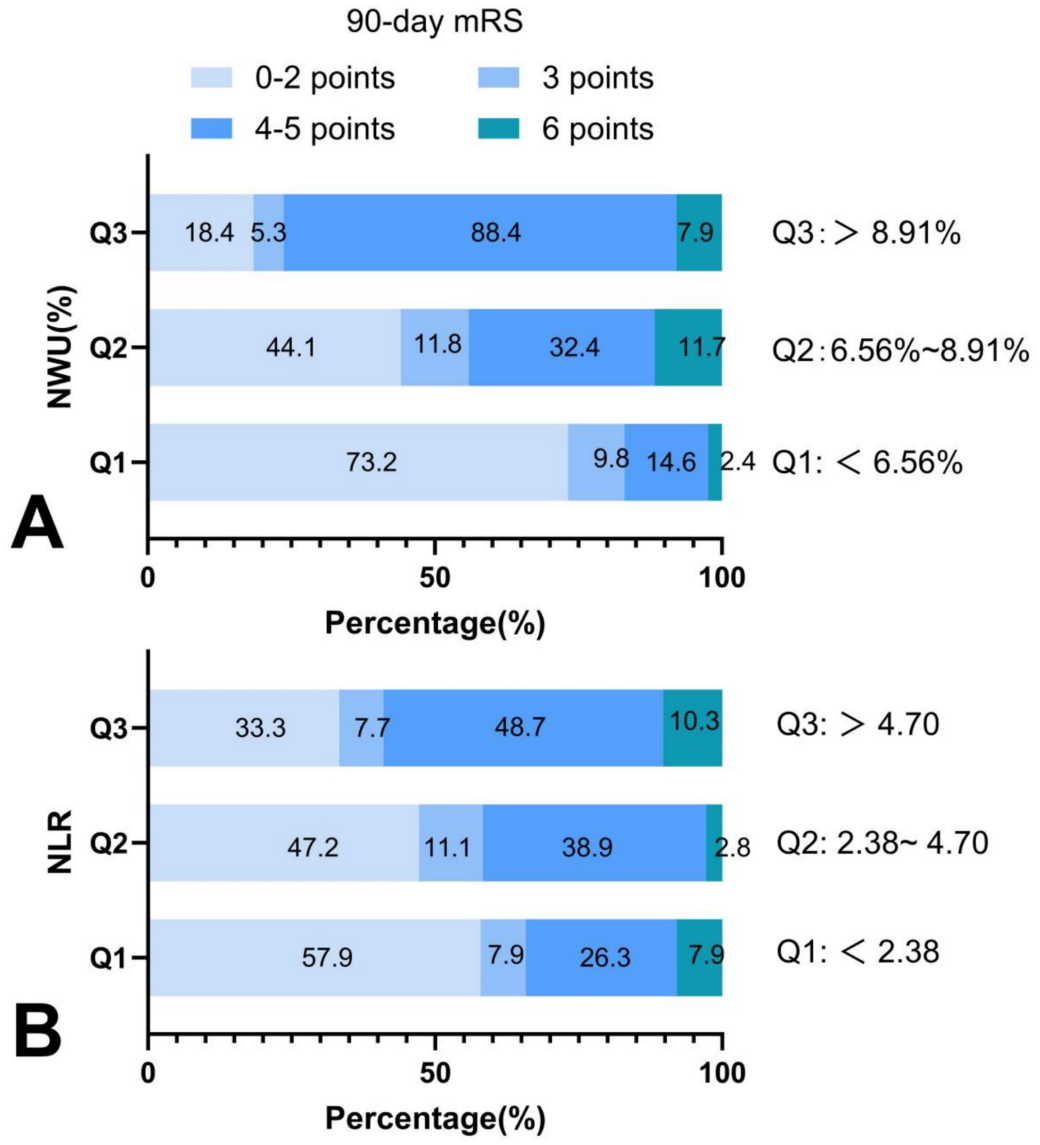
Distribution of 90-day mRS scores across NWU and NLR levels. **(A)** Along with increased NWU levels, the proportion of participants with good outcomes gradually decreased, and the proportion of participants with poor outcomes progressively increased; **(B)** with the increased NLR levels, the proportion of participants with good outcomes gradually decreased, and the proportion of participants with poor outcomes progressively increased. NWU indicates net water uptake; NLR, neutrophil-lymphocyte ratio; mRS, modified Rankin Scale.

When stratified by NWU tertiles (6.56, 8.91), along with increased NWU levels, the proportion of participants with good outcomes (mRS 0–2 points) gradually decreased (73.2, 44.1, and 18.4% in Q1, Q2, and Q3 groups, respectively), while the proportion of participants with severe disability (mRS 4–5 points) progressively increased (14.6, 32.4, and 68.4%, respectively). Mortality showed no dose-response relationship with NWU levels (Figure 4A).

When stratified by NLR tertiles (2.38, 4.70), the proportion of participants with good outcomes (mRS 0–2 points) gradually decreased with increased NLR levels (57.9, 47.2, and 33.3% in Q1, Q2, and Q3 groups, respectively), whereas the proportion of severe disability (mRS 4–5 points) progressively increased (26.3, 38.9, and 48.7%, respectively). Similarly, mortality demonstrated no dose-response relationship with NLR levels (Figure 4B).

### The association of NWU and NLR with 90-day outcomes in ordinal analysis

In the univariate ordinal logistic regression model, age, proportions of diabetes mellitus and previous stroke, Initial NIHSS, systolic blood pressure and diastolic blood pressure at admission, levels of glycosylated hemoglobin, NLR and NWU, and recanalization time were independently associated with 90-day functional outcome grading (Supplementary Table S1).

Included the above factors into a multivariate ordinal logistic regression model, both NLR (OR = 1.32, 95% CI = 1.12–1.56, *p* = 0.001) and MWU (OR = 1.29, 95% CI = 1.10–1.51, *p* = 0.002) were independently associated with the 90-day functional outcome grade. Glycated hemoglobin level (OR = 1.51, 95% CI = 1.06–2.17, *p* = 0.023) and recanalization time (OR = 1.01, 95% CI = 1.002–1.02, *p* = 0.015) were also independently associated with the functional outcome grade (Table 4).

**Table 4:** Clinical factors independently associated with 90-day outcomes grade in ALVOS patients with successful EVT recanalization.

Clinical factors	90-day functional outcome grade			
	Univariate model		Multivariate model	
	Crude OR	*p*-value	Adjusted OR	*p*-value
Age	1.03 (1.003–1.063)	0.03^*^		
Diabetes mellitus	2.15 (1.08–4.27)	0.028^*^		
Previous stroke	2.21 (1.04–4.72)	0.04^*^		
Initial NIHSS	1.15 (1.03–1.28)	0.012^*^		
SBP	1.03 (1.01–1.05)	0.001^*^		
DBP	1.04 (1.007–1.065)	0.015^*^		
HbA1c	1.38 (1.05–1.83)	0.022^*^	1.51 (1.06–2.17)	0.023^*^
NLR	1.19 (1.08–1.30)	<0.001^*^	1.32 (1.12–1.56)	0.001^*^
NWU	1.36 (1.17–1.60)	<0.001^*^	1.29 (1.10–1.51)	0.002^*^
Recanalization time	1.008 (1.001–1.02)	0.02^*^	1.01 (1.002–1.02)	0.015^*^

NIHSS indicates National Institutes of Health Stroke Scale; SBP, systolic blood pressure; DBP, diastolic blood pressure; HbA1c, glycosylated hemoglobin; NLR, neutrophil-lymphocyte ratio; NWU, net water uptake.

*p < 0.05 was considered statistically significant.

### ROC curve analysis

**Figure 5:**
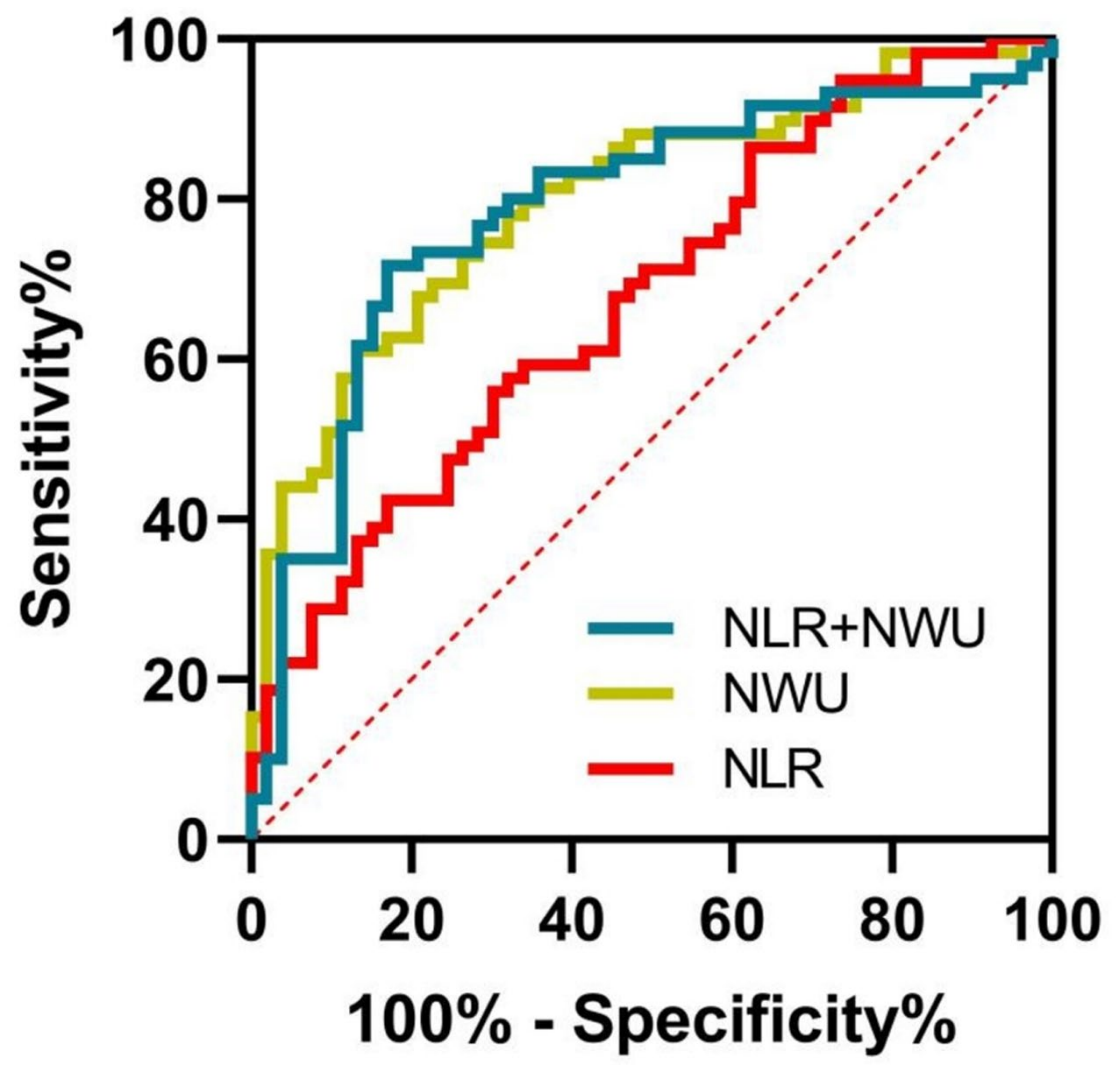
The indicative values of NWU and NLR for poor outcomes of participants. The combination of NWU and NLR had the highest AUC (0.800, 95% CI = 0.718–0.881, *p* < 0.001) in indicating poor outcome, followed by NWU (0.764, 95% CI = 0.674–0.855, *p* < 0.001) and NLR (0.662, 95% CI = 0.563–0.762, *p* = 0.003). NWU indicates net water uptake; NLR, neutrophil-lymphocyte ratio.

For indicating 90-day poor outcomes in ALVOS patients with successful EVT recanalization, NWU demonstrated an AUC of 0.764 (95% CI = 0.674–0.855, *p* < 0.001), with an optimal cut-off value of 8.00 (sensitivity 70.0%, specificity 81.1%). The NLR showed an AUC of 0.662 (95% CI = 0.563–0.762, *p* = 0.003), with a cut-off value of 3.90 (sensitivity 55.9%, specificity 69.8%). The combination of NWU and NLR further improved indicative value, yielding the highest AUC of 0.800 (95% CI = 0.718–0.881, *p* < 0.001) (Figure 5).

## Discussion

This study demonstrated that elevated levels of preoperative NWU on cranial CT and NLR were both independently and positively associated with 90-day poor outcomes in ALVOS patients with successful EVT recanalization. Notably, NWU exhibited a dose–response relationship with the risk of poor outcomes. Furthermore, the combination of NWU and NLR demonstrated promising indicative value for identifying patients at high risk of poor 90-day outcomes.

NWU in ischemic brain tissue has been recognized as a radiographic marker for quantifying cerebral parenchymal edema. Elevated NWU levels have been associated with malignant infarction or hemorrhagic transformation, thereby increasing the risk of poor outcomes after ischemic stroke (24). Several recent studies revealed that NWU levels were independently associated with 90-day poor outcomes in ALVOS patients undergoing EVT (25–27). However, whether preoperative NWU correlates with poor outcomes in ALVOS patients with successful EVT recanalization remains unclear. In this study, we found that preoperative NWU levels independently indicated 90-day poor outcomes in successfully recanalized ALVOS patients. Moreover, a significant dose–response relationship was observed between incremental NWU tertiles and the risk of poor outcomes. These findings suggest that preoperative NWU quantification could serve as a robust neuroimaging biomarker for outcome prognostication in successfully recanalized ALVOS populations.

In addition to NWU, our multivariate analysis identified NLR as another independent predictor of poor 90-day clinical outcomes in successfully recanalized ALVOS patients. After ischemic stroke, the inflammation activation and immune response rapidly arise along with the blood–brain barrier disruption (28). As a biomarker that can reflect systemic inflammatory status (29), NLR has been shown to be significantly correlated with outcomes in ischemic stroke patients who accepted EVT (30). This study further revealed that NLR was associated with clinical outcomes in ALVOS patients with successful EVT recanalization. Feng Y et al. also reported that NLR can predict poor functional outcomes in ALVOS patients with complete EVT recanalization (31), which was in accordance with this study. After the restoration of blood flow in the occluded blood vessels, ischemia–reperfusion injury takes place in the ischemic region, involving multiple pathophysiological processes, including pyroptosis, cell apoptosis, oxidative stress, and inflammatory response (32). In the first few hours after reperfusion, neutrophils are one of the first cells to infiltrate hypoxic tissue, causing damage to the blood–brain barrier and oligemic tissue surrounding the penumbra (33). In addition, some scholars reported that even if complete reperfusion was achieved, a substantial amount of stroke patients did not have satisfactory outcomes. Some of these patients might experience ineffective recanalization which was likely to be related to severe neuroinflammation (31). The above mechanism contributed to explain the impact of NLR on the clinical outcomes of ALVOS patients with successful EVT recanalization.

In this study, we employed restricted cubic spline analysis to investigate the associations of NWU and NLR with 90-day poor functional outcomes. The results revealed a significant nonlinear relationship between NWU and 90-day poor functional outcomes. When NWU was below 8%, the risk of poor functional outcome remained largely unchanged with increasing NWU. However, once NWU exceeded 8%, the risk of poor outcome increased markedly. In contrast, the nonlinear association between NLR and poor functional outcome at 90 days was not statistically significant. We subsequently conducted ROC analysis and found that the AUC of NWU indicating post-stroke disability was 0.764, while the NLR was 0.662. This validated that NWU may be more valuable than NLR in indicating poor clinical outcomes in ALVOS patients with successful EVT recanalization. In the subsequent analysis, the AUC of NWU combined with NLR in indicating post-stroke disability increased to 0.800, slightly higher than that of NWU alone, elucidating that NWU combined with NLR had more predictive value for post-stroke disability than a single indicator. This suggested that in clinical practice, the tests of preoperative NWU combined with NLR of ALVOS patients was potentially beneficial for predicting the clinical outcomes of these patients after successful recanalization, and could to some extent guide the clinical treatment strategies of these patients.

There are several pathophysiological links between NWU and NLR. The NLR is calculated as the neutrophil count divided by the lymphocyte count, both of which play important roles in maintaining blood–brain barrier (BBB) integrity. Neutrophils are among the first immune cells to respond to cerebral ischemia, infiltrating and accumulating around ischemic tissue, where they can exert neurotoxic effects and exacerbate brain injury (34). Additionally, neutrophils promote BBB breakdown through the release of matrix metalloproteinase-9 (MMP-9). A study has reported that elevated serum MMP-9 levels are positively correlated with BBB impairment (35). In contrast, lymphocytes—primarily T cells and B cells—exert neuroprotective effects following ischemic stroke by suppressing inflammation and maintaining BBB integrity (36). Thus, a high NLR reflects severe BBB disruption, which may lead to significant cerebral edema and consequently contribute to increased NWU levels.

In addition to NWU and NLR, we also found that recanalization time and glycated hemoglobin levels were independently associated with poor 90-day functional outcomes, consistent with previous studies (37, 38). A shorter recanalization time indicates more rapid reperfusion therapy, which facilitates the salvage of a larger proportion of the ischemic penumbra, thereby promoting functional recovery. Conversely, elevated glycated hemoglobin may contribute to neuronal injury through mechanisms such as mitochondrial dysfunction, lactate accumulation, and oxidative stress in neuronal cells, ultimately impairing functional outcomes (39). For patients with intracranial large vessel stenosis who are at risk of stroke, strict glycemic control is imperative.

The innovation of this study lies in the finding that the combination of NWU and NLR is a potential biomarker in predicting the 90-day outcomes in ALVOS patients with successful EVT recanalization. For ALVOS patients eligible for thrombectomy, the prompt calculation of both NWU and NLR levels prior to the procedure maybe helpful to predict the functional outcomes. A future prospective study is needed to validate our assumption in this study.

This study had some limitations. Firstly, this was a single-center retrospective study with a small sample size, and the results required further validation through multi-center prospective studies with large sample sizes. Secondly, although NWU was significantly correlated with the clinical outcomes of ALVOS patients with successful EVT recanalization, its measurement might be effected by the examiner’s head position and previous cerebral lesions. Therefore, the clinical application of NWU might be subject to some limitations. Thirdly, this study only focused on the preoperative biomarkers of the participants and did not dynamically monitor the evolution of NLR and NWU. The dynamic NWU combined with NLR could be incorporated into the regression model in future studies to more accurately predict the clinical outcomes of ALVOS patients with successful EVT recanalization.

## Conclusion

Both NWU and NLR were significantly correlated with the 90-day clinical outcomes of ALVOS patients with successful EVT recanalization. Compared with NWU or NLR alone, the combination of NWU and NLR was more valuable in indicating poor outcomes of ALVOS patients with successful EVT recanalization.

## Data Availability

The raw data supporting the conclusions of this article will be made available by the authors, without undue reservation.
