# Supplementary material for: Net water uptake combined with neutrophil-to-lymphocyte ratio predictive value after successful recanalization in acute large vessel occlusion stroke
Source: Front Neurol. 2025 Oct 13;16:1633967. doi: 10.3389/fneur.2025.1633967 (PMC12554569; doi:10.3389/fneur.2025.1633967)
Supplement: Supplementary file 1 [file Table_1.DOCX]

Supplementary Table S1 The associations of clinical characteristics with 90-day outcome grading in univariate ordinal logistic regression analysis.

| Clinical characteristics | 90-day functional outcome grading | | |
| --- | --- | --- | --- |
|  | OR | 95%CI | P-value |
| Male | 1.72 | 0.86–3.45 | 0.12 |
| Age | 1.03 | 1.003–1.063 | 0.03^*^ |
| Hypertension | 1.51 | 0.59–3.87 | 0.39 |
| Diabetes mellitus | 2.15 | 1.08–4.27 | 0.028^*^ |
| Atrial fibrillation | 1.65 | 0.83–3.27 | 0.15 |
| Smoking | 1.21 | 0.55–2.65 | 0.64 |
| Previous stroke | 2.21 | 1.04–4.72 | 0.04^*^ |
| Initial NIHSS | 1.15 | 1.03–1.28 | 0.012^*^ |
| SBP | 1.03 | 1.01–1.05 | 0.001^*^ |
| DBP | 1.04 | 1.007–1.065 | 0.015^*^ |
| Intravenous thrombolysis | 1.40 | 0.66–2.98 | 0.38 |
| TC | 0.97 | 0.66–1.41 | 0.66 |
| TG | 1.08 | 0.55–2.14 | 0.82 |
| LDL-C | 0.92 | 0.61–1.40 | 0.70 |
| HDL-C | 1.47 | 0.52–4.18 | 0.47 |
| BUN | 1.10 | 0.96–1.26 | 0.17 |
| Cr | 1.00 | 0.98–1.02 | 0.97 |
| Uric acid | 1.00 | 0.997–1.003 | 0.93 |
| HCY | 0.99 | 0.94–1.05 | 0.74 |
| HbA1c | 1.38 | 1.05–1.83 | 0.022^*^ |
| Serum lean protein | 0.98 | 0.88–1.10 | 0.98 |
| Lymphocyte count | 0.78 | 0.43–1.40 | 0.40 |
| Platelet count | 1.003 | 0.998–1.008 | 0.24 |
| NLR | 1.19 | 1.08–1.30 | <0.001^*^ |
| NWU | 1.36 | 1.17–1.60 | <0.001^*^ |
| DPT | 1.005 | 0.997–1.01 | 0.24 |
| Occlusion site |  |  |  |
| ICA | 1.04 | 0.49–2.22 | 0.92 |
| MCA trunk (M1-M2) | 0.96 | 0.45–2.05 | 0.92 |
| Number of retrieval attempts | 1.24 | 0.84–1.83 | 0.28 |
| Implant stent | 1.57 | 0.62–3.98 | 0.34 |
| Recanalization time | 1.008 | 1.001–1.02 | 0.02^*^ |

Abbreviations: NIHSS indicates National Institutes of Health Stroke Scale; SBP, systolic blood pressure; DBP, diastolic blood pressure; TC, total cholesterol; TG, triglyceride; LDL-C, low-density lipoprotein cholesterol; HDL-C, high-density lipoprotein cholesterol; Hcy, homocysteine; HbA1c, glycosylated hemoglobin; NLR, neutrophil-lymphocyte ratio; NWU, net water uptake; DPT, **door-to-puncture time; ICA,** internal carotid artery; MCA, middle cerebral artery.

* *p* < 0.05 was considered statistically significant
